# Combined Supplementation of Rumen-Protected Algae Powder and Rumen-Protected Choline Increases Docosahexaenoic Acid Content in Goat Milk

**DOI:** 10.3390/foods15142546

**Published:** 2026-07-19

**Authors:** Senyang Hu, Zihao Wang, Hejing Tang, Wenhua Jin, Jian He, Sufang Duan, Jianmin Zou, Yang Yang, Chang Liu, Pengjie Wang, Wei Tan, Ignatius Man-Yau Szeto, Genna Ba, Yinhua Zhu

**Affiliations:** 1 Key Laboratory of Precision Nutrition and Food Quality, Department of Nutrition and Health, China Agricultural University, No.17 Tsinghua East Road, Haidian District, Beijing 100083, China; 2 National Center of Technology Innovation for Dairy, Hohhot 010110, China; 3 Inner Mongolia Yili Industrial Group, Co., Ltd., Hohhot 010110, China; 4 Key Laboratory of Cattle and Sheep Milk and Meat Products Risk Control and Key Technology, State Administration for Market Regulation, Hohhot 010110, China

**Keywords:** docosahexaenoic acid, functional dairy products, lipid metabolism, rumen-protected technology, dairy goats

## Abstract

Docosahexaenoic acid (DHA) is an essential omega-3 polyunsaturated fatty acid with important health benefits. However, DHA enrichment in ruminant milk is limited by inefficient post-absorptive transport. This study evaluated whether combined supplementation of rumen-protected algae powder (RPA) and rumen-protected choline (RPC) enhances DHA enrichment in goat milk. Nine lactating dairy goats were assigned to three groups (*n* = 3/group) for 28 days: RPA alone, RPA + low-dose RPC (5 g/d), and RPA + high-dose RPC (10 g/d). Milk DHA content, bioconversion efficiency, serum biochemical parameters, and lipid profiles were analyzed. Compared with RPA alone, low- and high-dose RPC increased milk DHA content to 27.98 and 33.50 mg/100 mL, respectively, representing increases of 23.0% and 47.3%, and enhanced DHA bioconversion efficiency to 20.66% and 24.29% compared with 16.86% in the RPA group. RPC supplementation increased serum VLDL and triglyceride concentrations, and lipidomics revealed increased DHA-containing triglycerides (TG-DHA). These findings suggest that RPC may enhance DHA enrichment in goat milk by promoting VLDL-mediated DHA transport. Further studies with larger animal populations are required to confirm these effects. These findings contribute to a better understanding of nutritional regulation of DHA transfer and provide insights into strategies for developing DHA-enriched dairy products.

## 1. Introduction

Docosahexaenoic acid (DHA, 22:6 n-3) is a long-chain omega-3 polyunsaturated fatty acid with important functions in human health. As a structural component of neuronal and retinal membranes, it contributes to brain development, visual function, and cognitive performance [1,2], while also exhibiting anti-inflammatory and immunomodulatory activities associated with the prevention of cardiovascular, metabolic, and neurodegenerative diseases [3,4]. However, global DHA intake remains below recommended levels in many populations [5], highlighting the need for accessible dietary carriers capable of efficiently delivering DHA while maintaining its bioactivity.

Compared with conventional sources such as fish oil and algal oil, dairy products are attractive vehicles for DHA enrichment because of their wide consumer acceptance, suitability for daily consumption, and favorable bioavailability [6,7]. Enriched milk can be produced either by directly adding DHA-rich oils or by supplementing dairy animals with DHA-containing feeds, thereby allowing this fatty acid to be naturally incorporated into milk fat. The latter approach has attracted increasing attention because incorporation into the native lipid matrix may improve oxidative stability, sensory properties, and bioaccessibility [7,8,9]. Nevertheless, feed-to-milk transfer efficiency in ruminants remains relatively low, limiting the development of naturally enriched dairy products [10,11].

One major limitation is ruminal biohydrogenation, during which a large proportion of dietary DHA is converted into saturated fatty acids by rumen microorganisms [12]. Rumen-protected algae powder (RPA) has therefore been developed to increase ruminal bypass and intestinal availability. Previous studies have shown that this protected algal source increases milk DHA content [13,14,15]; however, increasing supplementation levels does not proportionally improve DHA conversion efficiency [16], suggesting that additional metabolic constraints exist beyond ruminal degradation. After intestinal absorption, long-chain fatty acids must be transported through the circulation and taken up by the mammary gland [10]. In ruminants, DHA uptake by the mammary gland primarily depends on triglyceride (TG)-bound DHA transported by chylomicrons and very low-density lipoproteins (VLDL) [17,18]. Because circulating concentrations of the latter are inherently low, the availability of lipoprotein carriers may constrain the delivery of TG-bound DHA to mammary tissue [19]. Enhancing hepatic lipid export may therefore provide an additional approach to improving feed-to-milk transfer.

Choline is a vitamin-like nutrient involved in phosphatidylcholine (PC) synthesis, which is essential for hepatic assembly and secretion of VLDL [20,21]. Rumen-protected choline (RPC) is widely used in ruminant nutrition to improve hepatic lipid metabolism and alleviate fatty liver [22,23]. Previous studies demonstrated that RPC supplementation promotes hepatic VLDL synthesis and increases circulating VLDL concentrations in ruminants [24,25,26]. These findings suggest that adding RPC to an RPA-containing diet could increase the availability of circulating lipid carriers and thereby facilitate DHA transfer into milk.

Although RPA and RPC have each been investigated separately, their combined use for improving the transfer of dietary DHA into milk has received little attention. To our knowledge, few controlled feeding studies have examined whether adding RPC to an established RPA supplementation regimen can further increase milk DHA content and bioconversion efficiency, or whether such changes are associated with alterations in circulating DHA-containing lipid species. Addressing this gap may clarify whether postabsorptive lipid transport, in addition to ruminal protection, can be targeted to improve DHA enrichment in ruminant milk.

Therefore, the objective of the present study was to evaluate the effects of combined RPA and RPC supplementation on lactation performance, milk composition, milk DHA content, and DHA bioconversion efficiency in lactating dairy goats. In addition, serum biochemical analysis and untargeted lipidomics were performed to investigate the mechanisms underlying RPC-mediated enhancement of DHA transport and deposition in milk.

## 2. Materials and Methods

### 2.1. Reagents

The RPA and RPC products used in the animal experiment were provided by King Techina Co., Ltd. (Hangzhou, China). RPA product contained 50% *Schizochytrium* microalgae powder, with a DHA content of 8.9% and a rumen bypass rate of 60%; the RPC product contained a choline content of 60% and a rumen bypass rate of 80%. Both products were coated with a microencapsulated sustained-release composite material. Standards for cis-4,7,10,13,16,19-docosahexaenoic acid methyl ester (D2659, 98%) and undecanoic acid methyl ester (U0250, 98%) were obtained from Sigma-Aldrich Chemical Co. (St. Louis, MO, USA). The goat serum VLDL content assay kit was supplied by Solarbio Technology Co., Ltd. (Beijing, China). All chromatography-related reagents were purchased from Thermo Fisher Scientific Inc. (Waltham, MA, USA). Other analytical-grade solvents were purchased from Macklin Co., Ltd. (Shanghai, China) and Aladdin Biotechnology Co., Ltd. (Shanghai, China).

### 2.2. Experimental Design and Dietary Treatments

All procedures involving animals were conducted in strict accordance with the guidelines of the Welfare and Animal Experiment Ethics Review Committee of China Agricultural University (Ethics Approval No.: AW62306202-5-04). The dairy goats were housed in individual stalls and fed a TMR with a concentrate-to-forage ratio of 25:75, which was adjusted as needed according to practical conditions. The composition and nutritional characteristics of the basal diet are presented in Table 1. Feed was offered twice daily, with experimental supplements administered during the morning feeding. RPA and RPC were premixed and incorporated into the basal diet. Goats were fed individually, with ad libitum access to water, and orts were collected 1 h after feeding.

**Figure 1:**
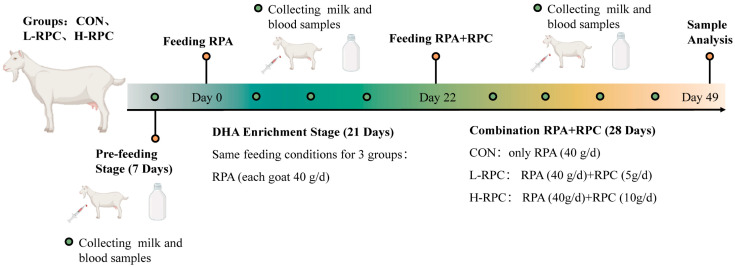
Experimental protocol flowchart for lactating goats during the entire feeding experimental period.

The overall experimental design is shown in Figure 1. Before the initiation of the feeding trial, all goats underwent a pre-adaptation period during which daily milk yield, dry matter intake, and body weight were recorded. Based on these baseline production parameters, animals were assigned to different dietary treatments using a balanced allocation approach to ensure comparable initial production performance among groups. The goats were allocated into three dietary treatment groups (*n* = 3 per group). This allocation procedure was implemented to minimize the influence of individual variation on treatment responses. Due to the nature of the feeding intervention, animal caretakers could not be blinded to dietary treatments; however, sample analysis and data processing were conducted according to standardized procedures.

The dosages of RPA and RPC were determined based on previous studies [14,25,27]. The formal experimental period consisted of two phases. Phase 1 was a 21-day algae powder supplementation period, during which all groups received the same diet (basal diet supplemented with 40 g/d RPA) to elevate and stabilize milk DHA levels. Phase 2 was a 28-day co-supplementation period, during which goats received different levels of RPA and RPC: the control group (CON) received the basal diet plus 40 g/d RPA; the low-dose RPC group (L-RPC) received the basal diet supplemented with 40 g/d RPA and 5 g/d RPC (0.2% of DMI); and the high-dose RPC group (H-RPC) received the basal diet supplemented with 40 g/d RPA and 10 g/d RPC (0.4% of DMI).

### 2.3. Sample Collection and Pretreatment

Daily feed refusals were collected and dried in a forced-air oven at 105 °C for 24 h to determine dry matter content, and dry matter intake was subsequently calculated.

Milk samples were collected during the pre-feeding period to serve as baseline measurements. During the formal experimental period, milk samples were collected twice weekly. At each sampling, individual milk yield was recorded, and the average yield was used for subsequent analyses. Milk samples were obtained 2 h after feeding, with morning and evening milk pooled proportionally into 50 mL centrifuge tubes and stored at −20 °C until analysis.

Blood samples were collected from the jugular vein during the pre-feeding period as base line samples. During the formal experiment, blood samples were collected once weekly, 2 h after the morning feeding. Approximately 3–5 mL of blood was collected per sampling into a clot activator Vacutainer tube (Becton Dickinson, Franklin Lakes, NJ, USA), immediately placed on ice, and promptly processed. Samples were allowed to clot at room temperature in darkness for 45 to 60 min before centrifugation at 3000× *g* for 15 min at room temperature, and the resulting supernatant was aliquoted into 1.5 mL microcentrifuge tubes, rapidly frozen in liquid nitrogen, and stored at −80 °C for subsequent analysis.

### 2.4. Nutritional Composition of Milk

The protein content in milk samples was determined according to a previously described method using the Kjeldahl nitrogen determination procedure [28]. Briefly, 10 mL of milk sample was transferred into a digestion tube, followed by the addition of 5 g of a K_2_SO_4_-CuSO_4_ catalyst mixture (*w*/*w*, 9:1) and 20 mL of concentrated H_2_SO_4_. The samples were digested at high temperature for 2 h using an automatic digestion system. Titration data were recorded, and the protein content of goat milk was subsequently calculated. The determination of milk fat content and total solids was conducted according to the method described by [29].

### 2.5. DHA Content and Bioconversion Efficiency

The DHA content in milk samples was determined using the acetyl chloride–methanol method described by Wang et al. [7] and Zou et al. [8], with minor modifications. Briefly, approximately 2 mL of goat milk was accurately measured and transferred into a 15 mL anaerobic fermentation tube, and freeze-dried for more than 48 h. Subsequently, 2 mL of toluene and 3.5 mL of acetyl chloride–methanol solution (1:9, *v*/*v*) were added. The tube was flushed with nitrogen gas, thoroughly mixed, and incubated in a water bath at 80 °C for 2 h. After incubation, the tubes were removed and cooled to room temperature. The reaction mixture was then transferred to a 50 mL centrifuge tube and washed twice with 4 mL of sodium carbonate solution (10%, *w*/*w*, aqueous). The washing solution was combined with the reaction mixture and thoroughly mixed. The mixture was centrifuged at 5000 r/min for 5 min, and 1 mL of the supernatant was collected for analysis. DHA content was determined using gas chromatography with flame ionization detection.

Chromatographic separation was performed on an SP-2560 capillary column (100 m × 0.25 mm i.d., 0.25 μm film thickness; Sigma-Aldrich, MO, USA). The injector and detector temperatures were set at 270 °C and 260 °C, respectively. The oven temperature program was as follows: an initial temperature of 130 °C held for 5 min, increased at a rate of 4 °C/min to 240 °C, and maintained at 240 °C for 20 min. Nitrogen (purity > 99.99%) was used as the carrier gas, with a split ratio of 100:1 and an injection volume of 1.0 μL. DHA was identified by comparing the retention time of sample peaks with that of an authentic DHA standard. Quantification was performed using a calibration curve constructed with DHA methyl ester standards. Methyl undecanoate (C11:0) was used as an internal standard to ensure analytical accuracy and precision. The analytical method for DHA determination was validated before sample analysis. Calibration curves were generated using DHA reference standards at a series of concentrations, showing good linearity within the tested range (R^2^ > 0.999). Quality-control samples were analyzed periodically throughout the analytical sequence to monitor instrument stability and analytical reproducibility. The bioconversion efficiency of DHA in goat milk was calculated using Equation (1):

(1) $$\mathrm{DHA}\;\mathrm{bioconversion}\;\mathrm{efficiency}\;(\%)\;=\;T_{milk}/T_{intake}\;\times \;100\%$$

where $T_{milk}$ represents the total DHA output in milk, and $T_{intake}$ represents the total DHA intake.

$T_{milk}$ was calculated as:
$T_{milk}\;$= $C_{milk}/100\times \;Y_{milk}$, where $C_{milk}$ is the DHA concentration in milk (mg/100 g), and $Y_{milk}$ is the milk yield (kg).
$T_{intake}$ was calculated as:
$T_{intake}\;$= $C_{algae}\;\times \;A_{algae}\;\times \;DMI$, where $C_{algae}$ is the DHA content in the RPA product (8.9 g/100 g), $A_{algae}$ is the supplementation level of algal product in the RPA (500 g/kg), and DMI is the actual dry matter intake (kg).

### 2.6. Serum Biochemical Analysis

Serum VLDL levels were determined using an ELISA-based method. Briefly, VLDL in plasma samples was selectively precipitated using a dextran sulfate-magnesium ion (DS-Mg^2+^) reagent. After centrifugation, the supernatant was collected, and the residual apolipoprotein B (ApoB) content in the supernatant was quantitatively measured by ELISA to indirectly estimate VLDL levels. Since VLDL particles are rich in ApoB, precipitation of VLDL results in a marked reduction in ApoB concentration in the supernatant. The extent of this reduction is proportional to the VLDL content in the original sample.

Other serum biochemical parameters, including alanine aminotransferase (ALT), aspar tate aminotransferase (AST), total protein (TP), globulin (GLB), albumin (ALB), TG, urea (UREA), creatinine (CREA), BHB, total cholesterol (CHOL), and non-esterified fatty acids (NEFA), were measured using a fully automated biochemical analyzer (Hitachi 7020, Hitachi Ltd., Tokyo, Japan). Serum biochemical parameters were measured using an automated biochemical analyzer according to the manufacturer’s instructions. Internal quality controls were included during analysis to monitor assay accuracy and precision.

### 2.7. Lipid Extraction and Lipidomics Analysis

Serum samples were subjected to lipidomic analysis using a high-performance liquid chromatography-mass spectrometry system, following previously described lipidomics protocols [7,8,9]. Briefly, 100 μL of serum was transferred into an Eppendorf tube, and 80 μL of methanol and 400 μL of methyl tert-butyl ether were added for lipid extraction. The mixture was vortexed for 60 s and sonicated at 5 °C and 40 kHz for 20 min. The solution was then incubated at −30 °C for 20 min, followed by centrifugation at 13,000× *g* and 2 °C for 10 min. A 350 μL aliquot of the supernatant was collected, vacuum-dried, and reconstituted in 100 μL of a solvent mixture (isopropanol:acetonitrile = 1:1). After 30 s, the sample was sonicated (40 kHz, 5 min, ice-water bath) and centrifuged (15,000× *g*, 20 min, 2 °C). The resulting supernatant was transferred to an injection vial, and 2 μL was injected into the UHPLC-MS system for lipidomic analysis.

Lipidomic analysis was performed using an ultra-high-performance liquid chromatography–mass spectrometry (UHPLC–MS) system equipped with a behh-c18 column (2.1 × 100 mm i.d., 1.6 μm particle size; Waters, Milford, MA, USA). The mobile phases consisted of water, acetonitrile, and isopropanol, with 10 mM ammonium formate added to both mobile phases A and B. Mobile phase A consisted of water/acetonitrile/isopropanol (5:3:2, *v*/*v*/*v*), whereas mobile phase B consisted of water/acetonitrile/isopropanol (1:9:90, *v*/*v*/*v*). The gradient elution program was as follows: mobile phase B was initially maintained at 10%, increased to 40% at 1 min, 75% at 3 min, and 100% at 6 min, maintained at 100% for 1 min, and then returned to 10% at 7 min. The total chromatographic run time was 10 min. The mass spectrometer was operated in both positive and negative electrospray ionization modes. The sheath gas flow rate was set at 60 psi, the auxiliary gas flow rate was set at 20 psi, and the auxiliary heater temperature was maintained at 370 °C. The ion spray voltage was set to +3000 V in positive mode and −3000 V in negative mode. MS/MS spectra were acquired using a data-dependent acquisition (DDA) mode with normalized collision energies of 20, 40, and 60 V. The mass range for MS detection was set from *m*/*z* 200 to 2000. Data were normalized before statistical analysis. Differential lipid metabolites were screened using appropriate statistical criteria with false discovery rate (FDR) correction for multiple comparisons. Selected lipid species were further evaluated based on biological relevance and pathway interpretation.

### 2.8. Statistical Analysis

All 9 goats completed the 56 d experimental period. No animals were withdrawn or excluded from the analysis. All goats received the intended dietary treatments as assigned, and no protocol deviations occurred. Thus, 3 animals per group were included in the final analysis for all outcome measures. No adverse events or side effects (e.g., diarrhea, feed refusal, or clinical illness) were observed in any group throughout the trial.

Experimental data were initially organized and processed using Microsoft Excel 2021. All experiments were conducted in triplicate, and results are presented as mean ± SD. Statistical analyses and data visualization were performed using SPSS 27.0 and GraphPad Prism 10.1.2. For comparisons among groups at a single time point, differences among groups were evaluated by one-way ANOVA followed by Duncan’s multiple range test, with significance set at *p* < 0.05. For lipidomic analyses, raw data were processed with Thermo Scientific LipidSearch V5.2.7 software for compound identification and characterization. Further statistical analyses and data visualization were conducted using the Majorbio Cloud online platform (https://cloud.majorbio.com).

Comparisons among treatment groups were performed separately at each predefined sampling time point using one-way ANOVA. Individual goats were considered the experimental units, and measurements obtained at different time points were not pooled as independent observations within a single analysis. The present analysis was not intended to test overall time effects or treatment × time interactions.

## 3. Results and Discussion

### 3.1. Production Performance of Dairy Goats

Table 2 summarizes the production performance of dairy goats during the combined supplementation of RPA and RPC. Neither dry matter intake, body weight, nor daily milk yield was affected by dietary treatment (*p* > 0.05), indicating that supplementation with RPA and RPC did not impair feed intake, body weight maintenance, or lactation performance. These results are consistent with previous reports showing that moderate supplementation of RPA or RPC has minimal effects on feed intake and milk production in ruminants [30,31]. In contrast, Zahra et al. [32] reported increased dry matter intake and milk yield in periparturient cows (body condition score ≥ 4.0) following RPC supplementation, potentially due to improved hepatic function and associated increases in appetite. Such inconsistencies among studies may reflect differences in physiological status, basal diet composition, supplementation level, or rumen protection efficacy. Although milk yield was numerically higher in the L-RPC and H-RPC groups than in the CON group on day 28, this difference was not statistically significant and should be considered an exploratory observation rather than evidence of a treatment-related effect.

### 3.2. Nutritional Composition of Goat Milk

Milk composition is a key indicator of milk quality and reflects the nutritional and metabolic status of dairy animals, thereby influencing both processing characteristics and nutritional value [33]. As shown in Table 3, no differences were observed among treatments in milk protein or total solids during the pre-feeding period or the RPA-only supplementation phase (*p* > 0.05). However, milk fat content decreased following RPA supplementation across all groups, declining from 3.83–3.96% during the pre-feeding period to 3.25–3.39%. This reduction is consistent with previous reports indicating that supplementation of unsaturated fatty acids, such as DHA, decreases milk fat content in ruminants [12,34]. This phenomenon has been associated with the formation of biohydrogenation intermediates that may interfere with mammary lipogenesis and milk fat synthesis [35]. In addition, in vitro studies by Zou et al. [11] showed that DHA supplementation (algal powder or algal oil) alters rumen microbial composition, particularly the relative abundance of genera such as *Prevotella* and *Ruminococcus*. Although rumen microbial composition was not evaluated in the present study, these findings provide a possible explanation for the reduced milk fat content observed following RPA supplementation.

During the combined supplementation of RPA and RPC, no differences were observed among treatments in milk protein or total solids (*p* > 0.05). However, milk fat content was greater in the H-RPC group than in the CON and L-RPC groups (*p* < 0.05), indicating that inclusion of RPC partially alleviated the reduction in milk fat induced by RPA supplementation. Choline, as a methyl donor and precursor of phosphatidylcholine, enhance hepatic VLDL synthesis, thereby promoting fatty acid transport to the mammary gland and improving their uptake and esterification for milk fat synthesis [25]. In addition, improved hepatic lipid metabolism may reduce fatty acid accumulation in the liver and facilitate their partitioning toward milk fat synthesis. Consistent with these findings, a meta-analysis by Arshad et al. [36] reported that RPC supplementation increases milk fat and protein contents, which agrees with the greater milk fat content observed in the H-RPC group in the present study.

### 3.3. DHA Content and Bioconversion Efficiency in Goat Milk

The transfer efficiency of DHA from the diet to milk is influenced not only by ruminal biohydrogenation but also by the circulating forms of DHA and the mammary gland’s uptake capacity [15]. As shown in Table 4, milk DHA content and bioconversion efficiency were monitored throughout the experimental period. During the pre-feeding stage, milk DHA content remained low (2.05–3.78 mg/100 mL), within the typical range reported for ruminant milk. During the RPA-only supplementation phase, both milk DHA content and bioconversion efficiency gradually increased over time, indicating increased DHA deposition in milk following RPA supplementation. Under RPA-only supplementation, milk DHA content ranged from 22 to 25 mg/100 mL, with individual bioconversion efficiency reaching a maximum of 19.05%, suggesting an increased transfer of dietary DHA into milk under the experimental conditions. By the third week, no significant differences in milk DHA content were observed among the three groups (*p* > 0.05), suggesting that DHA enrichment had largely stabilized by this stage.

**Figure 2:**
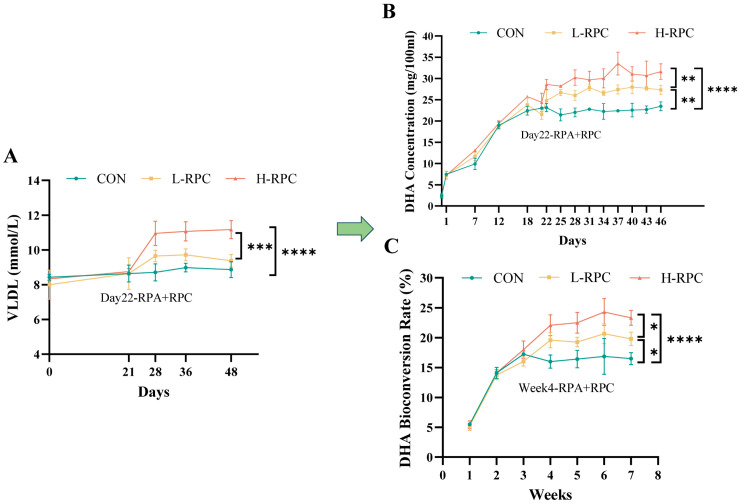
Enhanced DHA content and bioconversion rate in goat milk resulting from increased serum VLDL after combined RPA and RPC treatment. (**A**) Changes in serum VLDL concentrations in goats. (**B**) Dynamics of DHA concentration in goat milk. (**C**) Temporal changes in DHA bioconversion rate in goat milk. Data were presented as means ± SD, and analyzed by one-way ANOVA test. * *p* < 0.05, ** *p* < 0.01, *** *p* < 0.001, **** *p* < 0.0001. Error bars show the standard deviation.

Following the initiation of combined RPA and RPC supplementation, milk DHA content in the L-RPC and H-RPC groups increased significantly compared with the CON group (*p* < 0.05), with a parallel rise in DHA bioconversion efficiency. By the second week, the H-RPC group showed numerically higher DHA content and bioconversion efficiency than the other groups. By the third week, milk DHA concentrations reached their highest levels in the L-RPC and H-RPC groups (27.98 and 33.50 mg/100 mL, respectively), with maximum individual bioconversion efficiencies of 22.54% and 26.61%, significantly exceeding those of the CON group (22.75 mg/100 mL and 16.86%). These findings indicate that RPC supplementation provided an additional increase in milk DHA enrichment under a fixed RPA background. As shown in Figure 2A–C, temporal changes in serum VLDL paralleled those of milk DHA content and bioconversion efficiency.

### 3.4. Liver, Kidney, and Immune Function in Dairy Goats

Liver and kidney function are key indicators for evaluating the safety of dietary additives. ALT and AST are primarily localized in the cytosol and mitochondria of hepatocytes, and their increased activities in blood reflect hepatocellular damage. ALB, synthesized in the liver, serves as an indicator of hepatic synthetic capacity. Blood urea nitrogen and creatinine are commonly used to assess nitrogen metabolism and renal function, reflecting nitrogen utilization, hepatic urea cycling, and protein catabolism.

**Figure 3:**
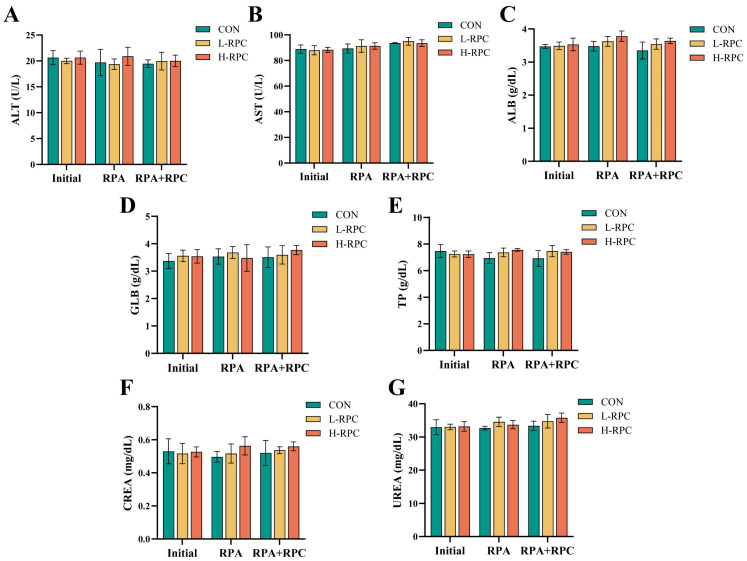
Serum biochemical parameters related to liver function, kidney function, and immune function in goats during the pre-feeding (Initial), algae powder supplementation (RPA), and combination (RPA + RPC) periods. Liver function: (**A**) ALT, (**B**) AST, (**C**) ALB. Immune function: (**D**) GLB, (**E**) TP. Kidney function: (**F**) CREA, (**G**) UREA. Data were presented as means ± SD, and analyzed by one-way ANOVA test. Error bars show the standard deviation.

As shown in Figure 3, combined supplementation of RPA and RPC did not affect serum indicators of liver function (ALT, AST, and ALB) or renal metabolism (UREA and CREA) compared with the CON group (*p* > 0.05). Previous studies have demonstrated the safety of RPA supplementation with respect to hepatic and renal function. For example, Mavrommatis et al. [37] reported that supplementation with 20, 40, or 60 g/d of *Schizochytrium* microalgae in lactating dairy goats had no adverse effects on blood biochemical parameters, with 20 g/d identified as an effective and sustainable strategy. Further analyses of rumen microbiota and enzyme activities revealed no increases in liver-associated enzymes. Similarly, studies evaluating RPC supplementation in periparturient ruminants have reported minimal effects on liver and kidney function. Zom et al. [25] and Swartz et al. [27] observed no differences in plasma liver function indicators (AST, ALT, and gamma-glutamyl transferase) between RPC-supplemented and control cows, indicating limited effects on hepatic inflammation and function. In dairy goats, Asadi et al. [30] found that supplementation with 6 g/d RPC did not affect liver enzyme activities (AST, ALT, and alkaline phosphatase) or serum CREA concentrations. Collectively, these findings support the safety of RPA and RPC supplementation in dairy goat diets with respect to liver and kidney function.

Serum globulin is an immunologically active protein that reflects immune function, whereas total protein can also serve as an indirect indicator of immune status. In the present study, no differences were observed in GLB or TP concentrations among treatments (*p* > 0.05), suggesting that RPA and RPC supplementation did not alter these measured serum indicators under the current experimental conditions. However, previous studies have suggested a positive role of choline in modulating immune responses. Lunsin et al. [38], in a systematic review of transition dairy cows, reported that RPC supplementation increased immunoglobulin G concentrations in colostrum and improved antioxidant status. Similarly, Asadi et al. [30] demonstrated that RPC supplementation in dairy goats increased immunoglobulin G and M concentrations in both does and their offspring, indicating enhanced immune function. The discrepancy between previous reports and the present findings may be related to differences in physiological status, as the goats in this study were in mid-lactation rather than the transition period, when metabolic and immune challenges are generally greater. Moreover, the present study evaluated only selected serum indicators, and whether RPC supplementation influences other aspects of immune function requires further investigation.

### 3.5. Lipid Metabolism in Dairy Goats

**Figure 4:**
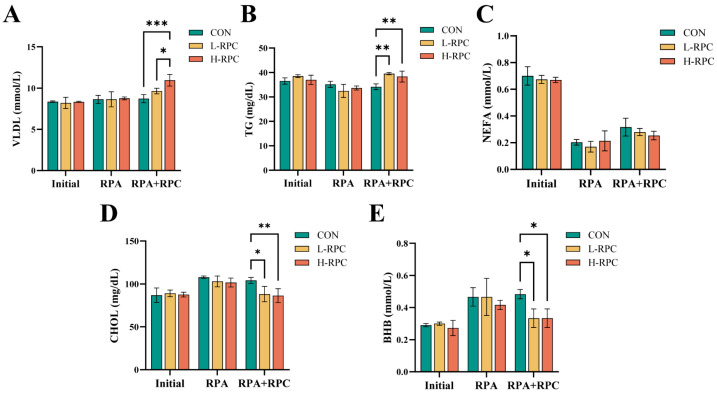
Serum lipid metabolism biochemical parameters in goats during the pre-feeding (Initial), algae powder supplementation (RPA), and combination (RPA + RPC) periods. (**A**) VLDL. (**B**) TG. (**C**) NEFA. (**D**) CHOL. (**E**) BHB. Data were presented as means ± SD, and analyzed by one-way ANOVA test. * *p* < 0.05, ** *p* < 0.01, *** *p* < 0.001. Error bars show the standard deviation.

DHA plays a central role in lipid metabolism, acting both as a regulatory factor and as a target for metabolic transport. In ruminants, the efficiency of DHA transport directly influences its enrichment in milk and is closely associated with hepatic VLDL synthesis, body fat mobilization, and circulating lipid metabolites. Serum lipid-related parameters in dairy goats are presented in Figure 4.

During the RPA-only supplementation phase, serum NEFA concentrations decreased compared with the pre-feeding period (*p* < 0.05), whereas TG and VLDL remained unchanged, and CHOL and BHB showed an increasing tendency. These responses are consistent with the physiological effects of PUFA-rich RPA. For example, DHA has been shown to reduce adipose tissue lipolysis and fatty acid release by activating peroxisome proliferator-activated receptor γ and inhibiting lipoprotein lipase activity, thereby lowering plasma NEFA concentrations [39]. This is in agreement with Mavrommatis et al. [40], who reported reduced plasma NEFA concentrations in dairy goats supplemented with *Schizochytrium* microalgae, supporting an anti-lipolytic effect of DHA. Despite the reduction in NEFA, BHB concentrations showed an increasing tendency, which may indicate a relative increase in incomplete oxidation of fatty acids in the liver. BHB is a key biomarker for ketosis in ruminants, with concentrations above 0.8 mmol/L indicating subclinical or clinical ketosis [41]. In the present study, all BHB values remained below 0.8 mmol/L, indicating a normal physiological status without evidence of ketosis. The increasing trend in CHOL may reflect the bidirectional regulation of cholesterol metabolism by DHA. Previous studies have shown that DHA can stimulate hepatic cholesterol synthesis while upregulating VLDL receptor expression to enhance clearance, potentially resulting in transient fluctuations in serum cholesterol under certain conditions [42]. The lack of changes in TG and VLDL during RPA supplementation suggests that RPA alone had limited effects on these circulating lipid transport indicators [43].

During the combined supplementation of RPA and RPC, serum VLDL and TG concentrations were greater in the L-RPC and H-RPC groups than in the RPA-only (CON) group (*p* < 0.05), whereas NEFA remained unchanged and CHOL and BHB were lower (*p* < 0.05). Once absorbed, choline serves as a precursor for PC synthesis in the liver, thereby promoting VLDL assembly and secretion. When choline supply is adequate, a greater proportion of hepatic triglycerides is exported as VLDL to peripheral tissues [44]. Because TG constitutes the core lipid component of VLDL particles, increased VLDL secretion is directly associated with higher circulating TG concentrations [18]. In addition, choline supplementation has been shown to alter plasma TG composition, increasing the proportion of PUFA-enriched TG [21]. The unchanged NEFA concentrations suggest that RPC supplementation did not further affect fatty acid mobilization under the present conditions, whereas the alterations in BHB and CHOL may reflect changes in hepatic lipid metabolism. However, these potential effects require further confirmation through direct measurements of hepatic lipid flux [25].

The increase in circulating VLDL-related lipids coincided with greater milk DHA enrichment and increased TG-DHA abundance after RPC supplementation. Although hepatic lipoprotein secretion and mammary DHA uptake were not directly evaluated, these findings suggest that altered circulating lipid transport may contribute to the enhanced incorporation of dietary DHA into milk. Therefore, VLDL-associated lipid transport represents a potential explanation for the observed improvement in DHA enrichment.

### 3.6. Serum Total Lipid Profile

**Figure 5:**
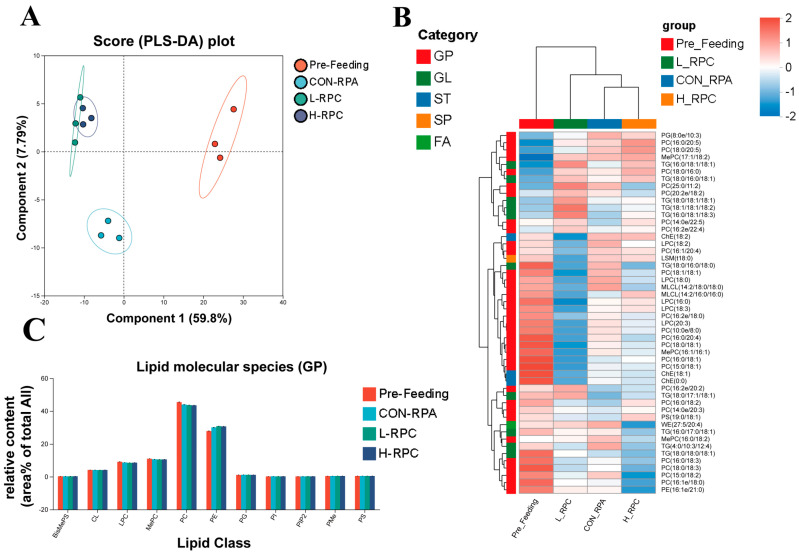
Differences in serum lipid composition among different treatment groups of goats during the combined RPA and RPC period. (**A**) Partial least squares discrimination analysis score plot of total lipids in different treatment groups. (**B**) Cluster analysis diagram of lipid components in serum of different treatment groups. Figure (**B**) shows the expression heatmap of serum lipid metabolites, where each row represents a metabolite, and the color indicates the relative expression level of that metabolite in the sample group. (**C**) Species and relative abundances of glycerophospholipid in serum of goats from different treatment groups. The horizontal coordinate represents different lipid subclasses, and the vertical coordinate represents the sum of the abundances of all lipid metabolites within each subclass. Groups: Pre-Feeding (initial baseline level); CON-RPA (RPA alone); L-RPC (RPA + low-dose RPC); H-RPC (RPA + high-dose RPC).

To elucidate the regulatory effects of combined RPA and RPC supplementation on lipid metabolism in dairy goats at the molecular level, serum samples were analyzed using untargeted lipidomics. Partial least squares discriminant analysis of the serum lipidome (Figure 5A) revealed clear separation among the Pre-Feeding, RPA-only (CON), and combined RPA and RPC supplementation (L-RPC and H-RPC) groups, with distinct clustering of samples within each group. This indicates significant differences in lipid metabolic profiles across feeding stages. Clustered heatmap analysis (Figure 5B) further illustrated the differences in lipid composition among groups. Notably, combined RPA and RPC supplementation markedly increased the relative abundance of glycerolipids (GL) in serum, which is consistent with the previously observed elevations in VLDL and TG concentrations. Glycerolipids, particularly TG, constitute the core lipid component of VLDL particles [18].

Notably, preliminary inspection of the clustered heatmap suggested a decreasing trend in glycerophospholipids (GP) following combined RPA and RPC supplementation, with levels appearing lower than those observed during the Pre-Feeding and RPA-only periods. However, quantitative analysis of total GP concentrations in serum indicated that the actual reduction was minor and did not reach statistical significance (Figure 5C). Further analysis of the GP subclass composition revealed a significant decrease in the relative abundance of PC, while the relative abundance of phosphatidylethanolamine (PE) significantly increased.

The marked decrease in PC can be reasonably explained from the perspective of VLDL synthesis and secretion. Choline, a key precursor for PC synthesis, increases hepatic PC synthesis upon RPC supplementation, thereby promoting the assembly and secretion of VLDL. As PC is an important structural lipid in the monolayer of VLDL, the increased secretion of VLDL is inevitably accompanied by a greater output of PC from the liver to peripheral tissues, leading to a decrease in serum PC levels [25]. In contrast, PE is not a major structural component of VLDL, and its consumption is comparatively lower. The significant increase in PE may be attributed to the following mechanisms: on one hand, the DHA-rich content of RPA can activate transcription factors such as peroxisome proliferator-activated receptor γ, upregulating the expression of genes involved in fatty acid and phospholipid synthesis, thereby promoting de novo PE synthesis [39]; on the other hand, the loading efficiency of PE into VLDL is lower than that of PC, leading to a relative retention of PE in the liver or passive release into the bloodstream, resulting in elevated serum PE levels.

### 3.7. Serum DHA-Containing Lipid Profile

**Figure 6:**
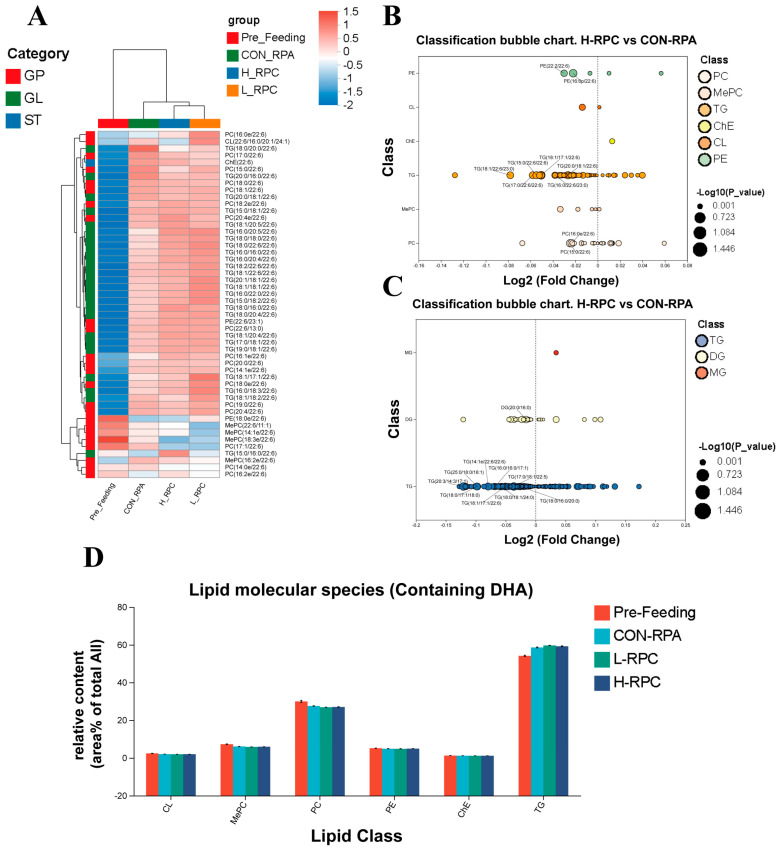
Differences in serum DHA-containing lipid composition among different treatment groups during the combined RPA and RPC period. (**A**) Cluster analysis diagram of DHA-containing lipid components in serum of different treatment groups. (**B**) Classification bubble chart of DHA-containing lipid molecules in serum between H-RPC and CON-RPA. (**C**) Classification bubble chart of glycerolipid molecules in serum between H-RPC and CON-RPA. (**D**) Species and relative abundances of DHA-containing lipid in serum of goats from different treatment groups. In the (**B**,**C**), each dot represents an individual lipid metabolite. The vertical coordinate represents the lipid subclass and different colors represent different lipid categories; the horizontal coordinate represents the fold change value of the expression differences between the two groups of metabolites, expressed as Log2 (Fold Change). Groups: Pre-Feeding (initial baseline level); CON-RPA (RPA alone); L-RPC (RPA + low-dose RPC); H-RPC (RPA + high-dose RPC).

To further investigate the mechanism by which combined RPA and RPC supplementation enhances milk DHA content, we analyzed the molecular composition of DHA-containing lipids in serum (Figure 6). Cluster analysis of DHA-containing lipids (Figure 6A) revealed that, compared with the Pre-Feeding period, RPA-only supplementation markedly increased the abundance of DHA-containing lipids in serum, indicating that DHA from RPA successfully entered systemic circulation and was incorporated into various lipid species, primarily GL and GP. Following combined RPA and RPC supplementation, the heatmap analysis showed a further enhancement in the expression of DHA-containing lipids, particularly TG-DHA and certain DHA-containing phosphatidylcholine (PC-DHA) and DHA-containing phosphatidylethanolamine (PE-DHA) species. These results indicate that RPC supplementation substantially promotes the incorporation of DHA into serum lipid molecules, with a pronounced effect on TG-DHA formation.

Figure 6B presents a bubble plot categorizing DHA-containing lipid species in serum across different treatment groups. This analysis further quantified the DHA-containing lipid subclasses that differed most significantly between the H-RPC and RPA-only groups, including TG, PC, PE, cholesteryl ester, cardiolipin, and methyl phosphatidylcholine. Among the top ten significantly altered lipid species, TG-DHA was predominant, followed by PC-DHA and PE-DHA. These findings are consistent with the cluster analysis, and the marked increase in TG-DHA abundance reflects an enhanced capacity of VLDL to incorporate and transport DHA. Figure 6C further presents a subclass-specific bubble plot for all GL species in serum. Compared with the RPA-only group, TG lipid species remained the most significantly altered component and were markedly elevated following combined RPA and RPC supplementation.

Figure 6D illustrates the composition and relative abundance of DHA-containing lipid species. The results indicated significant differences in TG-DHA and PE-DHA. The pronounced increase in TG-DHA is consistent with the previous findings, whereas the notable change in PE-DHA also warrants attention. PE is an essential phospholipid component of cell membranes and a precursor for PC synthesis, which can be converted to PC via phosphatidylethanolamine N-methyltransferase [23]. Research by Conway et al. [45] confirmed that choline deficiency significantly inhibits hepatic phosphatidylethanolamine N-methyltransferase activity, whereas choline supplementation restores enzyme activity and promotes PC synthesis. However, the rate of conversion from PE to PC may be limited by the rapid consumption of downstream PC for VLDL secretion, leading to the relative accumulation of PE-DHA. Taken together, the lipidomic results showed a shift toward TG-bound DHA in serum following RPC supplementation. This shift was associated with increased milk DHA content, but further tracer and tissue-level studies are required to determine whether it directly enhances mammary DHA uptake.

The results of this study may be applicable to lactating dairy goats under similar feeding conditions and supplementation levels. However, the small sample size (*n* = 3 per group) may limit the statistical power and generalizability of the findings. Further validation using larger animal populations is required to confirm the reproducibility of these effects. In addition, differences in lactation stage may influence lipid metabolism and DHA transfer efficiency, and therefore the extrapolation of these results to animals at different physiological stages or other production systems should be made with caution.

## 4. Conclusions

In summary, this study showed that combined supplementation of RPA and RPC enhanced DHA enrichment in goat milk without negatively affecting lactation performance. Compared with RPA alone, co-supplementation with RPC significantly increased milk DHA content and bioconversion efficiency, while partially alleviating the RPA-induced decline in milk fat content. Serum biochemical analyses indicated that RPC supplementation elevated circulating VLDL and TG concentrations, suggesting enhanced hepatic VLDL assembly and secretion. Untargeted serum lipidomics further revealed a marked increase in TG-DHA, which represented the predominant upregulated lipid subclass following combined supplementation.

These findings suggest that RPC may facilitate the circulating transport of TG-DHA to the mammary gland, thereby contributing to increased milk DHA content. However, the relatively small sample size, short experimental period, and use of a single dairy-goat population limit the statistical power and broader applicability of the findings. Therefore, the current results should be considered preliminary, and further validation using larger animal populations, different herds, and diverse lactation stages is warranted. Collectively, the findings highlight a promising nutritional strategy to overcome the limitation of low DHA transfer efficiency in ruminants and provide mechanistic and practical insights for the production of high-quality naturally DHA-enriched dairy products.

## Figures and Tables

**Table 1:** Ingredient and nutrient composition of the experimental diet.

Ingredient, DM%	Content	Nutritional Level, DM%	Content
Corn grain	20.00	Crude protein	14.70
Peanut vine	18.00	Neutral detergent fiber	56.01
Alfalfa hay	12.00	Acid detergent fiber	29.23
Corn silage	15.00	Starch	27.01
Concentrate supplement	25.00	Non-fibrous carbohydrates	35.43
Baking soda	2.00	Salt	0.41
Nutritious supplementary	3.00	Calcium	0.68
Wheat bran	5.00	Phosphorus	0.38
DHA content and choline in supplements		Ruminal passage rate (%)	Content (g/100 g)
RPA		60	8.90
RPC		80	60.40

**Table 2:** Effects of combining rumen-protected algae powder and rumen-protected choline on production performance of dairy goats.

Items ^1^	Treatment		
	CON	L-RPC	H-RPC
Feed intake, kg/d			
Day 1	2.26 ± 0.04	2.33 ± 0.06	2.34 ± 0.15
Day 14	2.30 ± 0.03	2.32 ± 0.15	2.33 ± 0.16
Day 28	2.35 ± 0.05	2.38 ± 0.12	2.36 ± 0.06
Body weight, kg			
Day 1	50.33 ± 2.39	48.50 ± 4.55	51.10 ± 2.69
Day 14	50.93 ± 3.29	49.50 ± 5.75	52.80 ± 1.66
Day 28	51.87 ± 4.24	49.97 ± 4.48	53.50 ± 2.97
Milk yield, kg/d			
Day 1	1.31 ± 0.05	1.28 ± 0.08	1.32 ± 0.11
Day 14	1.29 ± 0.06	1.27 ± 0.10	1.26 ± 0.10
Day 28	1.27 ± 0.02	1.37 ± 0.11	1.32 ± 0.10

^1^ Lactation performance of goats during the combined RPA and RPC phase. Data were recorded only during this period.

**Table 3:** Effects of combining rumen-protected algae powder and rumen-protected choline on milk composition.

Items ^1^	Treatment		
	CON	L-RPC	H-RPC
Milk fat, g/100 g			
Pre-Feeding	3.83 ± 0.22	3.96 ± 0.31	3.88 ± 0.19
RPA	3.25 ± 0.28	3.39 ± 0.14	3.33 ± 0.59
RPA + RPC	3.34 ± 0.12 ^b^	3.35 ± 0.17 ^b^	3.62 ± 0.12 ^a^
Milk protein, g/100 g			
Pre-Feeding	3.49 ± 0.22	3.66 ± 0.12	3.64 ± 0.22
RPA	3.62 ± 0.46	3.83 ± 0.61	3.74 ± 0.45
RPA + RPC	3.57 ± 0.60	3.75 ± 0.50	3.69 ± 0.36
Milk total solids, g/100 g			
Pre-Feeding	11.10 ± 1.22	11.90 ± 1.76	12.60 ± 1.65
RPA	12.61 ± 1.33	12.41 ± 1.55	12.73 ± 1.95
RPA + RPC	12.76 ± 1.46	11.98 ± 1.87	12.63 ± 0.67

^a,b^ Different letters represent statistically significant differences among different samples (lower case letters represent *p* ≤ 0.05). ^1^ Milk composition data from the Pre-Feeding, RPA alone, and RPA + RPC stages.

**Table 4:** Effects of combining rumen-protected algae powder and rumen-protected choline on the DHA content and bioconversion rate in the milk of dairy goats.

Items ^1^	Treatment		
	CON	L-RPC	H-RPC
DHA content, mg/100 mL and DHA bioconversion rate, %			
Pre-Feeding	2.31 ± 0.08 -	2.42 ± 0.14 -	2.39 ± 0.63 -
RPA alone			
Week 1	7.47 ± 0.12 5.52 ± 0.19%	7.02 ± 1.19 5.25 ± 0.77%	7.25 ± 0.78 5.43 ± 0.66%
Week 2	18.98 ± 0.81 14.12 ± 0.92%	18.61 ± 0.45 13.74 ± 0.70%	19.44 ± 0.64 14.09 ± 0.56%
Week 3	23.69 ± 1.41 17.25 ± 0.29%	22.56 ± 1.21 16.01 ± 0.77%	24.42 ± 2.15 18.01 ± 1.46%
RPA + RPC			
Week 1	22.04 ± 1.03 ^b^ 15.99 ± 1.12% ^b^	26.12 ± 1.68 ^a^ 19.57 ± 1.27% ^a^	29.69 ± 2.02 ^a^ 22.07 ± 1.72% ^a^
Week 2	22.26 ± 1.85 ^c^ 16.42 ± 1.46% ^c^	26.57 ± 0.54 ^b^ 19.26 ± 0.79% ^b^	30.03 ± 2.26 ^a^ 22.49 ± 1.73% ^a^
Week 3	22.75 ± 4.43 ^b^ 16.86 ± 2.99% ^b^	27.98 ± 1.54 ^ab^ 20.66 ± 1.63% ^ab^	33.50 ± 2.71 ^a^ 24.29 ± 2.24% ^a^
Week 4	22.57 ± 1.61 ^c^ 16.93 ± 1.65% ^c^	26.64 ± 2.18 ^b^ 19.77 ± 1.11% ^b^	31.62 ± 1.87 ^a^ 23.32 ± 1.25% ^a^

^a–c^ Different letters represent statistically significant differences among different samples (lower case letters represent *p* ≤ 0.05). ^1^ DHA content and bioconversion rate data from the Pre-Feeding, RPA alone, and RPA + RPC stages.

## Data Availability

The original contributions presented in the study are included in the article, further inquiries can be directed to the corresponding authors.

## Abbreviations

The following abbreviations are used in this manuscript:

ALB	albumin
ALT	alanine aminotransferase
ApoB	apolipoprotein B
AST	aspartate aminotransferase
CHOL	total cholesterol
CON	control group
CREA	creatinine
DHA	docosahexaenoic acid
GLs	glycerolipids
GLB	globulin
GPs	glycerophospholipids
H-RPC	high-dose rumen-protected choline group
L-RPC	low-dose rumen-protected choline group
NEFAs	non-esterified fatty acids
PC	phosphatidylcholine
PC-DHA	DHA-containing phosphatidylcholine
PE	phosphatidylethanolamine
PE-DHA	DHA-containing phosphatidylethanolamine
RPA	rumen-protected algae powder
RPC	rumen-protected choline
TG	triglyceride
TG-DHA	DHA-containing triglycerides
TP	total protein
UREA	urea
VLDL	very low-density lipoprotein
